# Supplementary material for: Determinants of COVID-19 Vaccine Uptake in The Netherlands: A Nationwide Registry-Based Study
Source: Vaccines (Basel). 2023 Aug 24;11(9):1409. doi: 10.3390/vaccines11091409 (PMC10537724; doi:10.3390/vaccines11091409)
Supplement: Supplementary file 1 [file vaccines-11-01409-s001.zip › vaccines-2538167-supplementary.pdf]

Supplementary material

S1 Long-term care recipients

To identify individuals receiving long-term care (LTC), two data sources were used. Firstly, start and end dates and types of LTC use were available within CBS. This is data from the CAK and includes LTC that is covered by the Long-term care act (WLZ) for which the LTC receiver pays a personal contribution to the CAK. An elaborate description of the database can be found (in Dutch) using the following URL: Gebwlztab: Personen Wlz-zorg (cbs.nl). Secondly, using the type of household variable in the personal records database (CBS) we were able to select individuals with 'institutional household' as type of household. A description of the personal records database and the variable type of household can be found (in Dutch) using the following URL: Gbapersoontab: Persoonskenmerken van personen in de BRP (cbs.nl)

In our analyses, we included three variables regarding LTC care distinguishing between residential and non-residential care and LTC for intellectual disabilities (residential and non-residential). For each variable, different selection methods were used:

1. LTC recipients, residential, nursing home: individuals were selected for this group if
    a. they received LTC care with profile VV5-8 (nursing and caring level 5-8) LTC and type of care 'care in kind'
    b. they lived in an institutional household according to the personal records database and received LTC with profile VV5-8
2. LTC recipients, residential, mentally impaired: individuals were selected for this group if
    a. they received LTC care with profile 'VG' (intellectually disabled) or 'LVG' (mildly intellectually disabled) and type of care 'care in kind'
    b. they lived in an institutional household according to the personal records database and received LTC with profile 'VG' or 'LVG'
3. LTC recipients, non-residential, mentally impaired: individuals were selected for this group if
    a. they received LTC care with profile 'VG' or 'LVG' and any type of care *other than* 'care in kind'
    b. they lived in any type of household *other than* institutional household according to the personal records database and received LTC with profile 'VG' or 'LVG'

S2 Standard random forest and ROC analysis

The standard RF predictor was constructed and assessed using a dataset of 400,000 people. This provided estimates of prediction accuracy such as the **PMC** (probability of misclassification, i.e. the probability of predicting an individual's status incorrectly), the **sensitivity** (the probability of an individual's status being predicted as a '1', i.e. as vaccinated, when its status is indeed '1'), the **specificity** (the probability of an individual's status being predicted as a '0', i.e. as unvaccinated (or not having provided informed consent to share their status), when its status is indeed '0'), and the ranking of the predictor variables according to their importance, which was measured as the average increase in (worsening of) the PMC that results from the replacement of the value of a variable by a randomly chosen value. As expected, the standard RF yielded a very high sensitivity and a comparatively low specificity because there were far more vaccinated (80%) than unvaccinated people and the default prediction rule aims at minimizing the PMC (as opposed to the sensitivity or specificity).

Figure S2.1 shows the results of an ROC analysis based on the RF. The algorithm's ordinary prediction rule consists of predicting an individual's outcome as a 1 (vaccinated) if and only if the ratio of estimated probabilities of that individual being a 1 to it being a 0 conditionally on the predictor variables is ≥1. If we replace this inequality by > ***c*** and we vary the value of the 'threshold' ***c*** over positive values other than 1 we create a family of predictors, each with its own, adjusted prediction rule, and hence with its own performance characteristics. In particular, by varying the value of the threshold we get a varying pair of sensitivity and fpr (false positive rate = 1 minus specificity) estimates, which constitute the ROC curve, shown in the left panel of Fig. 2. By looking at the performance indicators obtained by varying the threshold, as shown on the right panel of Figure S3.1, we can pick the value which yields similar estimates of sensitivity and specificity; in this case this is a c of 3.55 , with corresponding values of 70% sensitivity and specificity and an increased PMC of 30%, together with the area under the ROC curve, 0.76. The value of c was used to adjust the first RF, leading to the second, 'refined' RF, whose performance and variable importance were estimated by making predictions on the test data.

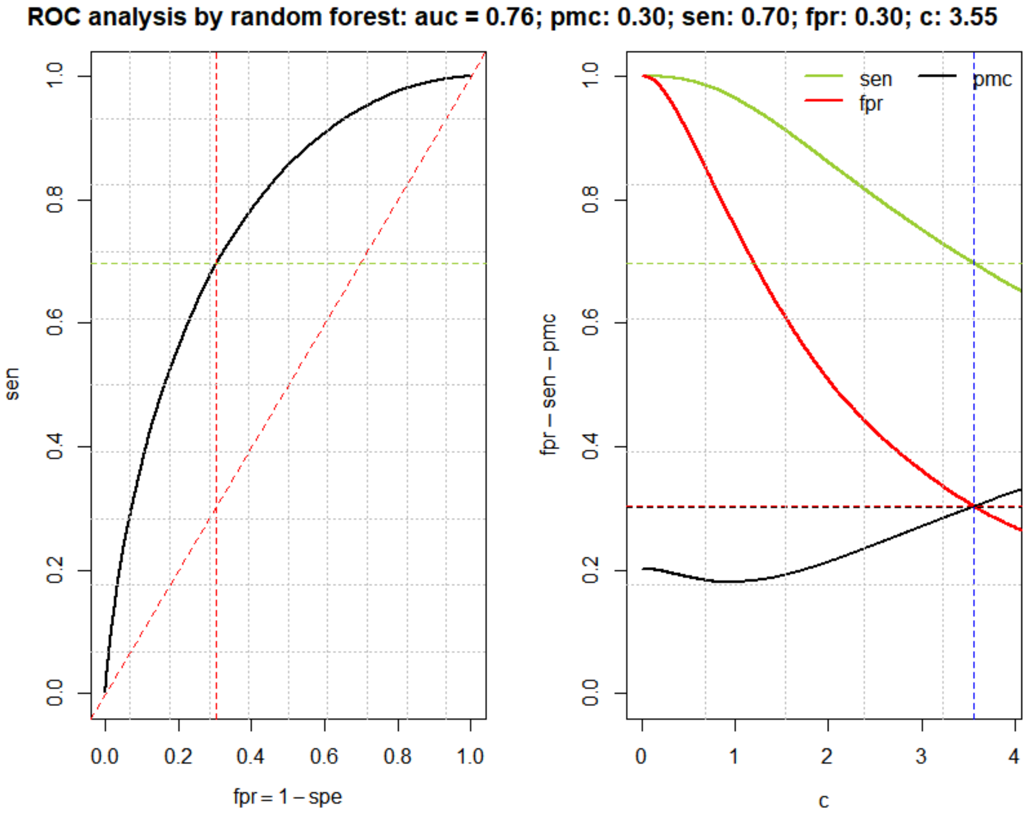

Figure S2.1 ROC analysis by random forest.

S3 Vaccine uptake - Bivariate analyses

All reported tables exclude frequencies below ten and all numbers and percentages are rounded to the nearest ten, to avoid personally identifiable information.

Table S3.1 Vaccine uptake by age group

	N	Uptake (N, %)
**Age group**		
18-35	4.012.330	2.683.820 (67)
36-50	3.260.110	2.526.670 (78)
51-66	3.845.610	3.339.460 (87)
67-79	2.211.910	2.024.530 (92)
80+	845.690	748.310 (88)

Table S3.2 Vaccine uptake per determinant by age group

	N	Uptake (N, %)
**Sex**		
Male	6.995.450	5.546.310 (79)
18-35	2.037.610	1.345.890 (66)
36-50	1.627.950	1.242.870 (76)
51-66	1.922.600	1.674.810 (87)
67-79	1.072.830	984.800 (92)
80+	334.460	297.950 (89)
Female	7.180.190	5.776.480 (80)
18-35	1.974.720	1.337.930 (68)
36-50	1.632.150	1.283.800 (79)
51-66	1.923.010	1.664.650 (87)
67-79	1.139.080	1.039.740 (91)
80+	511.230	450.370 (88)
**Education level**		
Primary education	680.660	482.560 (71)
18-35	115.660	59.860 (52)
36-50	165.170	102.470 (62)
51-66	243.680	185.030 (76)
67-79	140.210	120.700 (86)
80+	15.940	14.500 (91)
Prevocational secondary education-basic vocational programme (VMBO-b/k), lower secondary vocational training and assistant's training (MBO-1)	770.830	532.300 (69)
18-35	256.470	120.450 (47)
36-50	172.290	115.670 (67)
51-66	235.650	198.120 (84)
67-79	95.970	88.390 (92)

80+	10.460	9.670 (92)
Prevocational secondary education – theoretical and vocational programme (VMBO-g/t), the first three years of senior general secondary education (HAVO) and pre-university secondary education (VWO)	385.580	273.660 (71)
18-35	169.480	99.660 (59)
36-50	73.070	49.550 (68)
51-66	98.160	83.140 (85)
67-79	41.490	38.150 (92)
80+	3.370	3.150 (94)
Basic vocational training (MBO-2) and vocational training (MBO-3)	1.428.730	1.005.180 (70)
18-35	620.640	350.130 (56)
36-50	331.250	237.140 (72)
51-66	354.030	303.730 (86)
67-79	113.790	105.750 (93)
80+	9.020	8.450 (94)
Middle management and specialist education (MBO-4)	1.470.350	1.094.650 (74)
18-35	710.230	464.700 (65)
36-50	379.970	293.240 (77)
51-66	313.830	275.090 (88)
67-79	63.620	59.100 (93)
80+	2.690	2.530 (94)
Upper secondary education (HAVO/VWO)	898.270	694.220 (77)
18-35	568.380	421.100 (74)
36-50	133.520	100.780 (75)
51-66	156.840	136.040 (87)
67-79	36.940	33.890 (92)
80+	2.590	2.420 (94)
Hbo-, wo-bachelor	2.055.220	1.700.130 (83)
18-35	825.060	637.910 (77)
36-50	674.610	561.610 (83)
51-66	450.710	402.610 (89)
67-79	98.960	92.460 (93)
80+	5.880	5.550 (94)
Hbo-, wo-master, doctor	1.204.530	1.067.060 (89)
18-35	416.170	358.120 (86)
36-50	438.950	387.290 (88)
51-66	292.430	268.120 (92)
67-79	53.690	50.400 (94)
80+	3.290	3.140 (95)
Unknown	5.281.490	4.473.030 (85)
18-35	330.230	171.900 (52)
36-50	891.270	678.930 (76)
51-66	1.700.280	1.487.580 (87)
67-79	1.567.240	1.435.700 (92)
80+	792.460	698.910 (88)

**Country of origin**		
The Netherlands	10.660.430	9.045.520 (85)
18-35	2.703.240	2.030.150 (75)
36-50	2.269.290	1.874.450 (83)
51-66	3.074.150	2.743.040 (89)
67-79	1.883.420	1.746.340 (93)
80+	730.340	651.540 (89)
Turkey	333.090	177.880 (53)
18-35	133.490	48.110 (36)
36-50	110.090	67.880 (62)
51-66	67.890	47.480 (70)
67-79	16.800	11.160 (66)
80+	4.830	3.250 (67)
Morocco	294.480	119.200 (40)
18-35	120.060	25.420 (21)
36-50	94.790	43.960 (46)
51-66	55.760	34.490 (62)
67-79	17.900	11.770 (66)
80+	5.980	3.560 (60)
Surinam	304.370	191.400 (63)
18-35	99.830	44.970 (45)
36-50	83.550	52.020 (62)
51-66	85.850	65.980 (77)
67-79	28.710	23.400 (81)
80+	6.420	5.020 (78)
The Dutch Caribbean	136.230	70.510 (52)
18-35	60.260	23.610 (39)
36-50	35.650	18.850 (53)
51-66	28.320	18.970 (67)
67-79	10.310	7.820 (76)
80+	1.690	1.270 (75)
Indonesia	353.070	293.860 (83)
18-35	42.620	29.610 (69)
36-50	83.840	65.540 (78)
51-66	131.790	114.410 (87)
67-79	69.640	62.480 (90)
80+	25.170	21.830 (87)
Other Africa	227.900	134.870 (59)
18-35	106.320	50.560 (48)
36-50	66.990	43.690 (65)
51-66	45.340	33.650 (74)
67-79	7.960	6.010 (76)
80+	1.290	970 (75)
Other Asia	501.590	362.270 (72)
18-35	230.590	149.920 (65)
36-50	156.200	120.030 (77)

51-66	88.910	71.410 (80)
67-79	21.830	17.830 (82)
80+	4.070	3.080 (76)
Other America/Oceania	195.140	142.310 (73)
18-35	92.520	61.650 (67)
36-50	55.130	41.810 (76)
51-66	37.120	30.230 (81)
67-79	8.980	7.490 (83)
80+	1.400	1.140 (82)
Middle and eastern European countries within the EU	304.230	137.150 (45)
18-35	145.640	52.020 (36)
36-50	105.500	50.800 (48)
51-66	39.550	23.430 (59)
67-79	11.200	9.020 (80)
80+	2.340	1.890 (81)
GIPS countries	142.440	96.850 (68)
18-35	63.930	37.270 (58)
36-50	42.550	30.870 (73)
51-66	24.980	19.640 (79)
67-79	7.530	6.280 (83)
80+	3.450	2.800 (81)
Former or associated member states of the Commonwealth of Independent States	46.170	29.150 (63)
18-35	21.260	12.680 (60)
36-50	14.430	9.240 (64)
51-66	7.270	4.730 (65)
67-79	2.710	2.170 (80)
80+	510	340 (66)
Other countries of the EU	516.720	408.360 (79)
18-35	133.000	81.470 (61)
36-50	97.580	75.350 (77)
51-66	121.580	102.720 (84)
67-79	109.180	99.540 (91)
80+	55.390	49.280 (89)
Other European countries	159.780	113.460 (71)
18-35	59.570	36.400 (61)
36-50	44.530	32.190 (72)
51-66	37.120	29.290 (79)
67-79	15.740	13.230 (84)
80+	2.820	2.360 (83)
**Born in the Netherlands or abroad**		
Born in The Netherlands with both parents born in The Netherlands	10.660.430	9.045.520 (85)
18-35	2.703.240	2.030.150 (75)
36-50	2.269.290	1.874.450 (83)

51-66	3.074.150	2.743.040 (89)
67-79	1.883.420	1.746.340 (93)
80+	730.340	651.540 (89)
Born in the Netherlands with one parent born abroad	735.910	570.470 (78)
18-35	257.930	164.880 (64)
36-50	158.800	121.540 (77)
51-66	166.760	144.710 (87)
67-79	107.170	98.760 (92)
80+	45.260	40.590 (90)
Born in the Netherlands with two parents born abroad	527.880	241.560 (46)
18-35	337.560	113.810 (34)
36-50	124.550	71.370 (57)
51-66	48.760	41.090 (84)
67-79	13.000	11.730 (90)
80+	4.000	3.560 (89)
Born abroad with one parent born abroad	84.660	66.410 (78)
18-35	24.850	16.620 (67)
36-50	19.300	14.510 (75)
51-66	18.910	15.950 (84)
67-79	15.080	13.610 (90)
80+	6.520	5.720 (88)
Born abroad with two parents born abroad	2.053.270	1.305.630 (64)
18-35	653.310	331.700 (51)
36-50	657.390	420.250 (64)
51-66	508.020	369.170 (73)
67-79	181.470	143.370 (79)
80+	53.070	41.130 (78)
Born abroad with two parents born in the Netherlands	113.490	93.220 (82)
18-35	35.440	26.660 (75)
36-50	30.780	24.560 (80)
51-66	29.010	25.500 (88)
67-79	11.760	10.720 (91)
80+	6.500	5.780 (89)
**Socioeconomic position**		
In employment	6.984.360	5.618.950 (80)
18-35	2.470.560	1.735.130 (70)
36-50	2.324.820	1.911.280 (82)
51-66	2.161.520	1.947.610 (90)
67-79	26.030	23.650 (91)
80+	1.430	1.280 (90)
Self-employed	1.078.860	780.210 (72)
18-35	253.550	135.610 (53)
36-50	388.890	277.210 (71)

51-66	395.840	331.520 (84)
67-79	38.140	33.820 (89)
80+	2.440	2.070 (85)
Unemployment benefits (WW)	103.080	72.400 (70)
18-35	20.420	8.860 (43)
36-50	33.820	21.960 (65)
51-66	48.810	41.560 (85)
67-79	20	.
80+	.	.
Social assistance benefit	416.890	233.240 (56)
18-35	106.960	44.920 (42)
36-50	141.830	76.580 (54)
51-66	165.130	109.580 (66)
67-79	1.960	1.480 (76)
80+	1.020	680 (67)
Other benefits	266.950	178.090 (67)
18-35	117.520	68.330 (58)
36-50	70.180	46.990 (67)
51-66	78.100	61.820 (79)
67-79	380	320 (83)
80+	770	630 (82)
Disability benefit	521.330	387.210 (74)
18-35	55.310	25.850 (47)
36-50	140.340	94.440 (67)
51-66	325.650	266.890 (82)
67-79	30	.
80+	.	.
Pensioner	3.429.200	3.106.120 (91)
18-35	3.730	2.180 (59)
36-50	17.420	12.620 (72)
51-66	426.260	384.420 (90)
67-79	2.142.670	1.963.720 (92)
80+	839.120	743.180 (89)
Student	832.380	612.810 (74)
18-35	825.380	607.400 (74)
36-50	5.730	4.360 (76)
51-66	1.260	1.040 (82)
67-79	20	.
80+	.	.
Other/unknown	542.590	333.770 (62)
18-35	158.910	55.550 (35)
36-50	137.080	81.240 (59)
51-66	243.030	195.030 (80)
67-79	2.660	1.480 (56)
80+	910	470 (52)

**Personal income (percentiles)**		
0 till 10	706.770	488.830 (69)
18-35	447.550	312.420 (70)
36-50	86.120	54.440 (63)
51-66	139.530	112.140 (80)
67-79	16.980	5.330 (31)
80+	16.590	4.510 (27)
10 till 25	2.033.890	1.582.610 (78)
18-35	644.150	414.500 (64)
36-50	240.810	161.310 (67)
51-66	366.730	300.720 (82)
67-79	603.840	548.200 (91)
80+	178.360	157.880 (89)
25 till 50	3.481.510	2.695.920 (77)
18-35	864.710	519.310 (60)
36-50	640.570	452.280 (71)
51-66	811.500	668.440 (82)
67-79	803.200	729.430 (91)
80+	361.540	326.450 (90)
50 till 75	3.485.790	2.836.700 (81)
18-35	1.073.340	747.850 (70)
36-50	832.310	654.310 (79)
51-66	933.650	825.990 (88)
67-79	501.880	473.180 (94)
80+	144.620	135.370 (94)
75 till 90	2.091.710	1.787.130 (85)
18-35	515.240	398.710 (77)
36-50	675.350	560.580 (83)
51-66	696.980	633.000 (91)
67-79	167.180	159.740 (96)
80+	36.960	35.100 (95)
90 till 100	1.533.870	1.365.600 (89)
18-35	201.730	158.650 (79)
36-50	616.310	538.930 (87)
51-66	618.540	574.880 (93)
67-79	82.640	79.150 (96)
80+	14.640	13.990 (96)
Unknown	842.090	566.020 (67)
18-35	265.620	132.380 (50)
36-50	168.650	104.820 (62)
51-66	278.670	224.290 (80)
67-79	36.190	29.510 (82)
80+	92.980	75.010 (81)
**Household type**		
One-person household	3.075.860	2.344.020 (76)

18-35	878.160	569.020 (65)
36-50	499.110	336.070 (67)
51-66	728.590	584.370 (80)
67-79	586.680	516.430 (88)
80+	383.320	338.140 (88)
Unmarried couple without children	1.257.950	988.890 (79)
18-35	665.210	493.520 (74)
36-50	209.100	157.400 (75)
51-66	272.620	236.400 (87)
67-79	94.100	86.260 (92)
80+	16.910	15.310 (91)
Married couple without children	3.372.970	3.059.750 (91)
18-35	188.300	128.090 (68)
36-50	190.400	153.840 (81)
51-66	1.330.510	1.218.040 (92)
67-79	1.350.010	1.270.760 (94)
80+	313.760	289.020 (92)
Unmarried couple with children	1.052.990	799.390 (76)
18-35	374.200	242.570 (65)
36-50	495.060	399.570 (81)
51-66	171.380	147.210 (86)
67-79	10.930	8.980 (82)
80+	1.430	1.060 (74)
Married couple with children	4.101.880	3.248.490 (79)
18-35	1.392.250	966.940 (69)
36-50	1.523.520	1.245.210 (82)
51-66	1.071.830	940.260 (88)
67-79	97.260	82.590 (85)
80+	17.020	13.490 (79)
One-parent family	965.810	628.550 (65)
18-35	400.760	214.350 (53)
36-50	295.490	202.700 (69)
51-66	219.070	171.700 (78)
67-79	32.920	26.080 (79)
80+	17.580	13.720 (78)
Other household	101.440	63.750 (63)
18-35	65.660	38.610 (59)
36-50	13.470	7.330 (54)
51-66	14.380	11.160 (78)
67-79	6.080	5.130 (84)
80+	1.840	1.520 (82)
Institutional household	246.740	189.960 (77)
18-35	47.780	30.710 (64)
36-50	33.960	24.570 (72)
51-66	37.220	30.330 (81)
67-79	33.930	28.290 (83)

80+	93.840	76.060 (81)
**Household car ownership**		
Yes	11.113.920	9.101.130 (82)
18-35	2.848.490	1.929.370 (68)
36-50	2.669.600	2.122.330 (79)
51-66	3.283.050	2.904.110 (88)
67-79	1.838.870	1.712.610 (93)
80+	473.900	432.710 (91)
No	3.061.720	2.221.670 (73)
18-35	1.163.830	754.460 (65)
36-50	590.500	404.340 (68)
51-66	562.560	435.350 (77)
67-79	373.040	311.920 (84)
80+	371.790	315.600 (85)
**Employment sector**		
Activities of household as employer, undifferentiated goods and service producing activities of household for own use	31.030	21.420 (69)
18-35	6.320	2.730 (43)
36-50	9.150	6.050 (66)
51-66	11.360	8.960 (79)
67-79	3.790	3.320 (88)
80+	410	360 (90)
Agriculture, forestry and fishery	73.830	57.290 (78)
18-35	29.310	20.080 (69)
36-50	21.140	16.510 (78)
51-66	21.310	18.780 (88)
67-79	1.950	1.800 (93)
80+	130	.
Mining and quarrying	6.910	5.820 (84)
18-35	1.800	1.380 (77)
36-50	2.910	2.450 (84)
51-66	2.170	1.960 (90)
67-79	40	.
80+	.	.
Industry	717.940	586.020 (82)
18-35	205.920	145.140 (70)
36-50	240.560	196.370 (82)
51-66	265.370	238.770 (90)
67-79	5.850	5.520 (94)
80+	240	230 (94)
Electricity supply	29.730	25.260 (85)
18-35	8.870	6.780 (76)
36-50	10.960	9.300 (85)
51-66	9.610	8.910 (93)
67-79	280	260 (94)

80+	10	.
Water supply, sewage and waste management	35.770	28.660 (80)
18-35	9.270	6.130 (66)
36-50	12.390	9.890 (80)
51-66	13.900	12.440 (90)
67-79	200	190 (94)
80+	.	.
Construction	323.500	255.540 (79)
18-35	109.010	72.450 (66)
36-50	113.030	91.370 (81)
51-66	98.230	88.700 (90)
67-79	3.110	2.900 (93)
80+	130	.
Wholesale and retail trade	1.123.120	881.150 (78)
18-35	512.910	358.040 (70)
36-50	326.750	267.110 (82)
51-66	267.510	240.990 (90)
67-79	15.310	14.410 (94)
80+	640	600 (93)
Transportation and storage	363.240	280.800 (77)
18-35	113.780	71.740 (63)
36-50	111.120	86.260 (78)
51-66	128.980	113.990 (88)
67-79	9.180	8.640 (94)
80+	180	170 (94)
Accommodation and food services activities	261.770	198.610 (76)
18-35	163.730	116.810 (71)
36-50	54.940	43.730 (80)
51-66	40.580	35.700 (88)
67-79	2.420	2.280 (94)
80+	110	.
Information and communication	291.390	245.100 (84)
18-35	138.630	110.630 (80)
36-50	97.840	84.000 (86)
51-66	53.790	49.420 (92)
67-79	1.090	1.020 (93)
80+	40	.
Financial services	264.170	230.000 (87)
18-35	70.100	55.320 (79)
36-50	103.530	90.310 (87)
51-66	82.900	77.120 (93)
67-79	7.010	6.650 (95)
80+	620	590 (95)
Real estate activities	65.700	55.230 (84)
18-35	19.260	14.060 (73)
36-50	23.850	20.370 (85)

51-66	20.720	19.020 (92)
67-79	1.690	1.600 (94)
80+	190	180 (93)
Professional scientific and technical activities	515.870	440.230 (85)
18-35	223.000	179.200 (80)
36-50	166.110	143.960 (87)
51-66	119.250	109.970 (92)
67-79	7.230	6.850 (95)
80+	280	260 (93)
Administrative and support service activities	740.260	513.200 (69)
18-35	367.700	223.950 (61)
36-50	196.570	139.810 (71)
51-66	163.090	137.330 (84)
67-79	12.710	11.930 (94)
80+	190	180 (94)
Public administration and defence	529.030	455.280 (86)
18-35	142.170	110.050 (77)
36-50	183.370	157.170 (86)
51-66	202.020	186.640 (92)
67-79	1.450	1.400 (97)
80+	20	.
Education	529.830	459.890 (87)
18-35	185.110	149.120 (81)
36-50	178.860	156.640 (88)
51-66	162.420	150.880 (93)
67-79	3.380	3.200 (95)
80+	60	.
Human health and social work activities	1.348.300	1.113.670 (83)
18-35	509.860	377.020 (74)
36-50	402.870	340.760 (85)
51-66	429.630	390.340 (91)
67-79	5.870	5.480 (93)
80+	80	.
Arts, entertainment and recreation	106.330	87.350 (82)
18-35	49.420	37.710 (76)
36-50	27.650	22.930 (83)
51-66	27.540	25.070 (91)
67-79	1.640	1.570 (96)
80+	90	80 (89)
Other service activities	116.860	94.510 (81)
18-35	45.700	32.260 (71)
36-50	35.600	29.780 (84)
51-66	33.460	30.520 (91)
67-79	2.000	1.880 (94)
80+	90	.
Activities of extraterritorial organisations and bodies	1.090	800 (73)

18-35	220	150 (68)
36-50	400	280 (71)
51-66	460	360 (77)
67-79	10	.
80+	.	.
Other/unemployed/unknown	6.699.980	5.286.970 (79)
18-35	1.100.270	593.090 (54)
36-50	940.520	611.630 (65)
51-66	1.691.310	1.393.600 (82)
67-79	2.125.700	1.943.610 (91)
80+	842.190	745.040 (88)
**Urbanisation**		
Not urbanised	2.267.080	1.869.340 (82)
18-35	534.040	373.250 (70)
36-50	494.820	392.120 (79)
51-66	721.400	632.820 (88)
67-79	390.800	359.620 (92)
80+	126.020	111.530 (88)
Hardly urbanised	2.243.130	1.882.930 (84)
18-35	538.900	384.790 (71)
36-50	522.880	428.280 (82)
51-66	648.070	578.700 (89)
67-79	386.890	359.970 (93)
80+	146.400	131.180 (90)
Moderately urbanised	2.553.650	2.108.270 (83)
18-35	635.300	436.490 (69)
36-50	599.820	482.760 (80)
51-66	726.450	644.130 (89)
67-79	430.150	399.600 (93)
80+	161.940	145.290 (90)
Strongly urbanised	3.600.060	2.854.700 (79)
18-35	969.800	621.040 (64)
36-50	840.940	647.600 (77)
51-66	970.810	842.780 (87)
67-79	580.300	531.760 (92)
80+	238.210	211.530 (89)
Extremely urbanised	3.510.730	2.607.110 (74)
18-35	1.333.780	868.060 (65)
36-50	801.410	575.810 (72)
51-66	778.710	640.920 (82)
67-79	423.720	373.530 (88)
80+	173.120	148.790 (86)
Unknown	990	440 (44)
18-35	500	180 (36)
36-50	240	100 (41)

51-66	180	110 (61)
67-79	60	40 (77)
80+	.	.
**Medical risk groups**		
Low medical risk	10.598.450	8.259.750 (78)
18-35	3.577.840	2.385.010 (67)
36-50	2.708.720	2.084.480 (77)
51-66	2.743.830	2.367.020 (86)
67-79	1.216.950	1.110.960 (91)
80+	351.120	312.290 (89)
Intermediate medical risk	3.216.720	2.749.800 (85)
18-35	399.380	273.340 (68)
36-50	488.490	389.270 (80)
51-66	976.410	860.680 (88)
67-79	891.360	819.930 (92)
80+	461.090	406.570 (88)
High medical risk	360.480	313.250 (87)
18-35	35.110	25.480 (73)
36-50	62.910	52.920 (84)
51-66	125.370	111.760 (89)
67-79	103.600	93.640 (90)
80+	33.490	29.460 (88)
**Long term care recipients, residential, nursing home**		
No	14.066.530	11.232.240 (80)
18-35	4.012.270	2.683.780 (67)
36-50	3.259.660	2.526.300 (78)
51-66	3.840.720	3.335.270 (87)
67-79	2.188.690	2.004.750 (92)
80+	765.200	682.160 (89)
Yes	109.110	90.550 (83)
18-35	60	50 (81)
36-50	450	370 (82)
51-66	4.890	4.190 (86)
67-79	23.220	19.790 (85)
80+	80.490	66.160 (82)
**Long term care recipients, residential, mentally impaired**		
No	14.100.410	11.260.710 (80)
18-35	3.984.460	2.662.280 (67)
36-50	3.242.510	2.511.740 (77)
51-66	3.825.040	3.321.600 (87)
67-79	2.204.090	2.017.940 (92)
80+	844.310	747.140 (88)
Yes	75.230	62.090 (83)

18-35	27.870	21.540 (77)
36-50	17.600	14.930 (85)
51-66	20.560	17.860 (87)
67-79	7.820	6.590 (84)
80+	1.390	1.170 (84)
**Long term care recipients, non-residential, mentally impaired**		
No	14.149.170	11.303.980 (80)
18-35	3.993.510	2.670.900 (67)
36-50	3.255.850	2.523.510 (78)
51-66	3.843.000	3.337.370 (87)
67-79	2.211.190	2.023.940 (92)
80+	845.620	748.260 (88)
Yes	26.470	18.820 (71)
18-35	18.820	12.920 (69)
36-50	4.260	3.170 (74)
51-66	2.610	2.090 (80)
67-79	720	590 (83)
80+	70	50 (76)
**Voting proportions political movements (%)**		
**Progressive liberal**		
0-0.1	1.458.190	1.113.830 (76)
18-35	386.960	237.550 (61)
36-50	328.030	240.330 (73)
51-66	413.890	347.260 (84)
67-79	239.630	213.130 (89)
80+	89.690	75.550 (84)
0.1-0.2	8.902.660	7.139.080 (80)
18-35	2.301.410	1.493.560 (65)
36-50	2.031.010	1.570.240 (77)
51-66	2.530.710	2.211.210 (87)
67-79	1.481.490	1.365.810 (92)
80+	558.040	498.270 (89)
0.2-0.3	2.635.610	2.126.450 (81)
18-35	795.040	551.320 (69)
36-50	642.250	512.170 (80)
51-66	666.320	581.160 (87)
67-79	373.450	341.140 (91)
80+	158.560	140.660 (89)
0.3-0.4	1.042.600	828.770 (79)
18-35	453.500	340.020 (75)
36-50	235.450	184.540 (78)
51-66	211.780	179.520 (85)
67-79	105.840	93.840 (89)

80+	36.030	30.850 (86)
0.4-0.5	135.420	114.110 (84)
18-35	74.860	61.170 (82)
36-50	23.070	19.240 (83)
51-66	22.690	20.160 (89)
67-79	11.440	10.570 (92)
80+	3.360	2.980 (89)
Unknown	1.160	560 (48)
18-35	560	210 (38)
36-50	310	150 (48)
51-66	220	150 (66)
67-79	70	50 (78)
80+	10	.
**Right-wing liberal**		
0-0.1	373.280	245.010 (66)
18-35	157.910	94.170 (60)
36-50	91.290	58.280 (64)
51-66	80.420	58.910 (73)
67-79	33.880	26.410 (78)
80+	9.790	7.240 (74)
0.1-0.2	5.449.320	4.123.990 (76)
18-35	1.797.910	1.149.040 (64)
36-50	1.231.000	899.710 (73)
51-66	1.353.570	1.132.140 (84)
67-79	771.610	688.840 (89)
80+	295.230	254.260 (86)
0.2-0.3	7.101.440	5.876.040 (83)
18-35	1.764.450	1.222.390 (69)
36-50	1.638.170	1.316.970 (80)
51-66	2.042.290	1.812.370 (89)
67-79	1.197.490	1.112.150 (93)
80+	459.050	412.160 (90)
0.3-0.4	1.244.820	1.072.320 (86)
18-35	290.730	217.470 (75)
36-50	298.130	250.530 (84)
51-66	367.320	334.310 (91)
67-79	207.720	196.010 (94)
80+	80.920	74.000 (91)
0.4-0.5	5.620	4.880 (87)
18-35	780	540 (70)
36-50	1.220	1.030 (85)
51-66	1.780	1.580 (89)
67-79	1.160	1.080 (93)

80+	690	650 (93)
Unknown	1.160	560 (48)
18-35	560	210 (38)
36-50	310	150 (48)
51-66	220	150 (66)
67-79	70	50 (78)
80+	10	.
**Right-wing Christian**		
0-0.1	13.501.210	10.833.030 (80)
18-35	3.821.890	2.574.950 (67)
36-50	3.107.460	2.419.810 (78)
51-66	3.662.560	3.190.390 (87)
67-79	2.105.720	1.933.000 (92)
80+	803.590	714.880 (89)
0.1-0.2	359.010	278.500 (78)
18-35	95.340	59.940 (63)
36-50	81.040	60.680 (75)
51-66	98.680	83.980 (85)
67-79	59.720	53.500 (90)
80+	24.230	20.400 (84)
0.2-0.3	169.960	120.090 (71)
18-35	49.670	27.450 (55)
36-50	38.160	26.120 (68)
51-66	45.170	36.170 (80)
67-79	26.330	22.290 (85)
80+	10.630	8.050 (76)
0.3-0.4	48.250	31.370 (65)
18-35	15.310	7.960 (52)
36-50	11.710	7.380 (63)
51-66	12.510	9.440 (75)
67-79	6.400	5.020 (78)
80+	2.330	1.570 (67)
0.4-0.5	42.880	23.380 (55)
18-35	14.350	5.820 (41)
36-50	9.720	5.120 (53)
51-66	10.860	7.110 (66)
67-79	5.730	4.020 (70)
80+	2.220	1.320 (59)
0.5-0.6	21.750	8.720 (40)
18-35	8.030	2.040 (25)
36-50	5.190	1.970 (38)
51-66	5.160	2.770 (54)
67-79	2.530	1.540 (61)

80+	830	400 (48)
0.7+	400	160 (39)
18-35	180	50 (26)
36-50	100	40 (43)
51-66	100	50 (50)
67-79	20	10 (58)
80+	10	.
Unknown	32.180	27.550 (86)
18-35	7.570	5.620 (74)
36-50	6.720	5.550 (82)
51-66	10.580	9.540 (90)
67-79	5.460	5.150 (94)
80+	1.850	1.690 (91)
**Christian middle**		
0-0.1	5.671.140	4.325.370 (76)
18-35	1.899.850	1.228.340 (65)
36-50	1.349.300	1.002.580 (74)
51-66	1.401.260	1.184.530 (85)
67-79	742.270	666.560 (90)
80+	278.450	243.370 (87)
0.1-0.2	6.818.410	5.633.990 (83)
18-35	1.674.670	1.157.410 (69)
36-50	1.532.780	1.228.230 (80)
51-66	1.965.300	1.738.410 (88)
67-79	1.186.740	1.099.500 (93)
80+	458.920	410.440 (89)
0.2-0.3	1.371.420	1.103.080 (80)
18-35	355.040	238.790 (67)
36-50	307.190	239.030 (78)
51-66	389.670	337.710 (87)
67-79	230.780	210.420 (91)
80+	88.740	77.150 (87)
0.3-0.4	276.010	229.180 (83)
18-35	71.300	51.340 (72)
36-50	61.410	49.410 (80)
51-66	79.080	69.780 (88)
67-79	46.480	42.900 (92)
80+	17.740	15.760 (89)
0.4-0.5	37.490	30.600 (82)
18-35	10.910	7.730 (71)
36-50	9.110	7.280 (80)
51-66	10.080	8.890 (88)
67-79	5.570	5.110 (92)

80+	1.830	1.600 (88)
Unknown	1.160	560 (48)
18-35	560	210 (38)
36-50	310	150 (48)
51-66	220	150 (66)
67-79	70	50 (78)
80+	10	.
**Right-wing conservative**		
0-0.1	1.122.670	875.120 (78)
18-35	479.340	350.980 (73)
36-50	254.890	194.940 (76)
51-66	232.860	193.770 (83)
67-79	115.110	100.800 (88)
80+	40.480	34.630 (86)
0.1-0.2	5.939.710	4.798.900 (81)
18-35	1.691.030	1.157.600 (68)
36-50	1.388.260	1.096.670 (79)
51-66	1.576.110	1.378.350 (87)
67-79	916.020	840.240 (92)
80+	368.290	326.060 (89)
0.2-0.3	6.238.150	4.973.740 (80)
18-35	1.621.070	1.043.330 (64)
36-50	1.423.280	1.093.280 (77)
51-66	1.777.640	1.547.310 (87)
67-79	1.032.020	948.690 (92)
80+	384.140	341.130 (89)
0.3-0.4	815.300	631.040 (77)
18-35	205.270	122.990 (60)
36-50	179.320	131.760 (73)
51-66	241.060	205.290 (85)
67-79	139.510	126.700 (91)
80+	50.140	44.300 (88)
0.4-0.5	32.580	23.490 (72)
18-35	8.310	4.430 (53)
36-50	7.940	5.470 (69)
51-66	10.210	8.300 (81)
67-79	4.730	4.140 (88)
80+	1.390	1.150 (83)
0.5-0.6	26.080	19.930 (76)
18-35	6.750	4.280 (63)
36-50	6.100	4.410 (72)
51-66	7.500	6.290 (84)
67-79	4.470	3.910 (87)

80+	1.250	1.040 (83)
Unknown	1.160	560 (48)
18-35	560	210 (38)
36-50	310	150 (48)
51-66	220	150 (66)
67-79	70	50 (78)
80+	10	.
**Progressive left-wing**		
0-0.1	467.900	339.290 (73)
18-35	137.840	80.980 (59)
36-50	107.150	74.860 (70)
51-66	125.820	102.030 (81)
67-79	69.990	59.950 (86)
80+	27.100	21.480 (79)
0.1-0.2	5.206.530	4.340.030 (83)
18-35	1.272.160	895.490 (70)
36-50	1.218.320	988.790 (81)
51-66	1.516.920	1.350.910 (89)
67-79	870.730	810.120 (93)
80+	328.410	294.730 (90)
0.2-0.3	6.603.020	5.195.710 (79)
18-35	1.839.480	1.175.010 (64)
36-50	1.509.220	1.147.940 (76)
51-66	1.783.490	1.539.630 (86)
67-79	1.055.650	965.120 (91)
80+	415.190	368.020 (89)
0.3-0.4	1.660.520	1.278.500 (77)
18-35	674.370	476.470 (71)
36-50	367.600	274.760 (75)
51-66	359.590	299.830 (83)
67-79	189.950	168.110 (89)
80+	69.000	59.320 (86)
0.4-0.5	236.510	168.710 (71)
18-35	87.930	55.670 (63)
36-50	57.520	40.190 (70)
51-66	59.570	46.910 (79)
67-79	25.530	21.180 (83)
80+	5.980	4.770 (80)
Unknown	1.160	560 (48)
18-35	560	210 (38)
36-50	310	150 (48)
51-66	220	150 (66)
67-79	70	50 (78)

80+	10	.

S4 Vaccine uptake in the Netherlands per neighbourhood

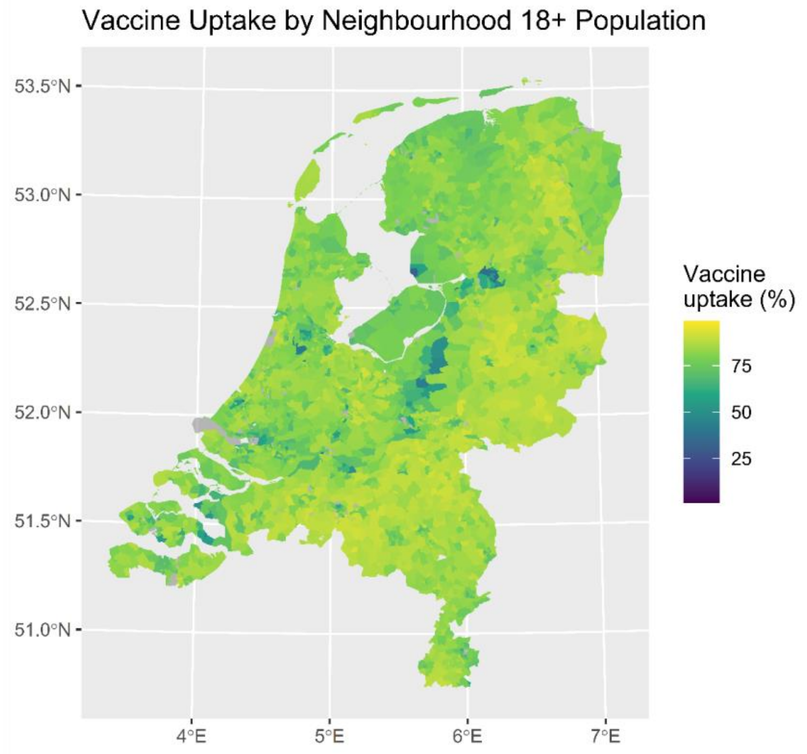

Figure S4.1 Vaccine uptake (%) in the Netherlands per neighbourhood in the population of 18 years and older

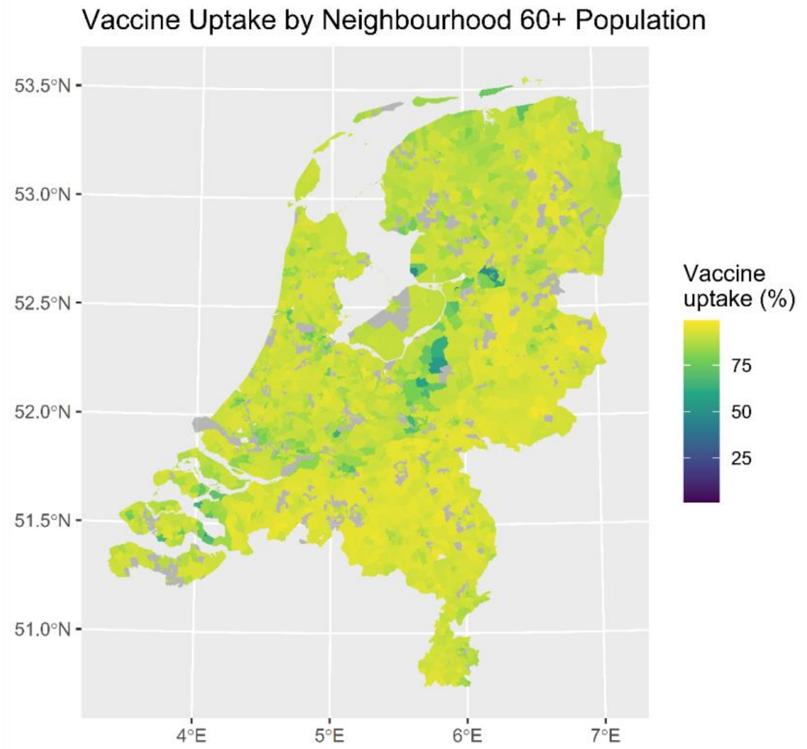

Figure S4.2 Vaccine uptake (%) in the Netherlands per neighbourhood in the population

of 60 years and older

Note: the grey areas indicate neighbourhoods with frequencies <10 and were therefore excluded
